# Second Language Learning in Older Adults: Effects on Brain Structure and Predictors of Learning Success

**DOI:** 10.3389/fnagi.2021.666851

**Published:** 2021-06-03

**Authors:** Jonna Nilsson, Rasmus Berggren, Benjamín Garzón, Alexander V. Lebedev, Martin Lövdén

**Affiliations:** ^1^Aging Research Center, Karolinska Institute (KI), Stockholm University, Stockholm, Sweden; ^2^Department of Physical Activity and Health, Swedish School of Sport and Health Sciences, Stockholm, Sweden; ^3^Department of Psychology, University of Gothenburg, Gothenburg, Sweden; ^4^Department of Clinical Neuroscience, Karolinska Institute, Stockholm, Sweden

**Keywords:** foreign language learning, gray matter (GM), white matter (WM), old age, plasticity

## Abstract

It has previously been demonstrated that short-term foreign language learning can lead to structural brain changes in younger adults. Experience-dependent brain plasticity is known to be possible also in older age, but the specific effect of foreign language learning on brain structure in language-and memory-relevant regions in the old brain remains unknown. In the present study, 160 older Swedish adults (65–75 years) were randomized to complete either an entry-level Italian course or a relaxation course, both with a total duration of 11 weeks. Structural MRI scans were conducted before and after the intervention in a subset of participants to test for differential change in gray matter in the two groups in the inferior frontal gyrus, the superior temporal gyrus, and the hippocampus, and in white matter microstructure in the superior longitudinal fasciculus (SLF), inferior longitudinal fasciculus (ILF), fronto-occipital fasciculus, and the hippocampal (HC) section of the cingulum. The study found no evidence for differential structural change following language training, independent of achieved vocabulary proficiency. However, hippocampal volume and associative memory ability before the intervention were found to be robust predictors of vocabulary proficiency at the end of the language course. The results suggest that having greater hippocampal volume and better associative memory ability benefits vocabulary learning in old age but that the very initial stage of foreign language learning does not trigger detectable changes in brain morphometry in old age.

## Introduction

Structural brain changes following short-term foreign language learning have been demonstrated previously in younger adults. Language training has been associated with morphometric brain changes in cortical regions that previously have been linked to language and memory functions, including changes in cortical thickness and gray matter probability in the inferior frontal gyrus (Martensson et al., 2012; Hosoda et al., 2013), cortical thickness in the superior temporal gyrus (Martensson et al., 2012), and gray matter probability and volume of the hippocampus (Martensson et al., 2012; Bellander et al., 2016). Structural brain change has also been demonstrated in the white matter following second language training in younger adults, often in tracts that connect important cortical language areas (Schlegel et al., 2012; Hosoda et al., 2013). The majority of language training regimes that have reported structural brain changes in T1-weighted MR images have lasted for a few months to a year (Li et al., 2014), but diffusion MRI has demonstrated microstructural gray matter change after less than 1 h of word learning (Hofstetter et al., 2017). However, the exact language experience (e.g., extent, content, immersion) required for anatomical changes to occur is largely unknown.

In contrast, the timing of second language learning is known to be important, with the typical finding being a higher achieved proficiency if the language is learned in childhood relative to in adulthood (Johnson and Newport, 1989). Whilst perfect mastery of the second language is less likely to be achieved in an adult learner, foreign language learning can be achieved to a high level also in adulthood (Hartshorne et al., 2018). It has even been proposed that foreign language training for older adults could serve as a tool to improve cognition by engaging the extensive language network and potentially mitigate age-related cognitive decline (Antoniou et al., 2013). However, evidence for general cognitive benefits following language training in older adults has so far been unconvincing (Bak et al., 2016; Ramos et al., 2017; Ware et al., 2017) and we have recently shown that an entry-level Italian course did not confer any general cognitive advantage relative to relaxation training (Berggren et al., 2020).

Whilst the cognitive effects of language training may be limited to the language acquisition itself, the effect of language training in the old brain is nevertheless important for our understanding of experience-dependent neuroplasticity and the brain processes involved in foreign language learning. In the visuomotor domain, it has been shown that older participants demonstrate similar albeit smaller gray matter changes in response to juggling practice compared to younger adults, evidencing that experience-dependent structural brain change occurs also in old age (Draganski et al., 2004; Boyke et al., 2008). However, there has been no previous intervention study that has investigated the effect of language training on brain structure in older individuals.

The primary aim of the present study was therefore to investigate the effect of language training on brain structure in older adults in several language-and memory-related gray matter regions and white matter tracts, all specified *a priori*. Based on theoretical accounts of the neural basis of language processing and previous demonstrations of gray matter changes following language training in younger individuals, we hypothesized that older participants without any previous knowledge of the Romance languages would demonstrate differential change in the inferior frontal gyrus, the superior temporal gyrus, and in the hippocampal (HC) volume following language training relative to relaxation training (Hickok and Poeppel, 2007; Davis and Gaskell, 2009; Martensson et al., 2012). We also hypothesized that gray matter change in the same regions would be associated with achieved vocabulary proficiency at the end of language training, as indicated by a comprehensive vocabulary test. The number of gray matter regions of interest was limited to four to maintain an acceptable level of statistical sensitivity to detect effects even after correcting for multiple tests.

For the white matter, we hypothesized that language training would result in a differential change in tracts that connect important language and memory regions, incorporating both the articulatory-dorsal and the semantic-ventral language processing streams (Friederici and Gierhan, 2013). As such, differential change was hypothesized in the white matter microstructure, measured by fractional anisotropy (FA) and mean diffusivity (MD), in the arcuate fasciculus, including the fibers extending to the superior longitudinal fasciculus (SLF), and in the inferior longitudinal fasciculus (ILF), including fibers that extend to the fronto-occipital fasciculus (Assaf and Pasternak, 2008; Catani and Thiebaut de Schotten, 2008; Friederici, 2009). Finally, given the importance of memory in language learning, white matter microstructure in the portion of the cingulum that connects with the hippocampus was also hypothesized to show the differential change (Duff and Brown-Schmidt, 2012). As with the gray matter predictions, we also hypothesized that white matter change in the same tracts would be associated with achieved vocabulary proficiency at the end of language training.

A complementary aim of the study was to investigate possible predictors of achieved vocabulary proficiency in participants who completed the language training. Given previous demonstrations of associations between white matter microstructure and language learning success (Floel et al., 2009; Lopez-Barroso et al., 2013; Qi, 2015; Ripolles et al., 2017), we hypothesized that white matter microstructure in the already specified language- and memory-related tracts, before the intervention, would be predictive of vocabulary learning success. In a more detailed specification, structural equation modeling (SEM) was used to investigate associative memory and hippocampal volume before the language intervention as predictors of vocabulary learning success. Evidence of age-related reductions of hippocampal volume and associative memory performance suggests that these factors may be important limiting factors for second language learning in older adults (Naveh-Benjamin, 2000; Van Petten, 2004; Raz et al., 2004). Given the requirement in the vocabulary learning to encode, store and retrieve associations between foreign words and their native counterparts, associative memory ability was hypothesized to be an important predictor of vocabulary learning success. The hippocampus has been proposed as critical for associative memory with particular relevance for language learning and was therefore also hypothesized to be an important predictor (Ullman, 2004; Suzuki, 2008; Duff and Brown-Schmidt, 2012). In addition, hippocampal volume was hypothesized to also be related to associative memory at baseline, and the two were expected to predict shared as well as unique aspects of vocabulary learning success.

Although the language system is traditionally modeled as being left-lateralized (Hickok and Poeppel, 2007), we did not restrict any of our hypotheses to the left hemisphere. This was motivated by functional imaging evidence that implicates a widespread language network in both hemispheres (Glasser and Rilling, 2008; Price, 2010) as well as the possibility that the structural changes resulting from second language learning may not be lateralized in the same way as the neural basis of the first language (Hosoda et al., 2013; Qi, 2015). Analyses, therefore, included the specified regions of interest in both hemispheres. Second language learning was implemented as an 11-week long entry- level Italian course with biweekly teacher-led classes and homework, which is comparable to previous studies in young adults that have demonstrated structural brain change (Hosoda et al., 2013; Bellander et al., 2016) and represents a feasible and thereby ecologically valid undertaking for the older population.

The present work represents the first investigation of experience-dependent structural brain change following language training in older adults. All hypotheses were pre-registered and all results are reported in direct relation to these hypotheses (gray matter hypotheses^1^; white matter hypotheses^2^).

## Materials and Methods

### Participants

Healthy older adults, aged 65–75 years, were recruited to the study through ads in a local newspaper. All participants were native Swedish speakers with no substantial prior knowledge of any of the Romance languages. All participants had at least working knowledge of English and self-rated their prior knowledge in Italian as either “nonexistent” or “very poor.” All participants were cognitively intact, as indicated by a MMSE score of 25 or above. Participants were randomly allocated to attend an Italian course or a relaxation course in a 6:5 ratio, due to practical reasons of scheduling. The maximum group size for both courses was 12 participants and the minimum group size was 10 at course start and nine at study completion. Sex, age, and associative memory performance were used as stratifiers in the randomization procedure. Randomization occurred once participants had consented to participate and completed the first day of cognitive testing in the pretest phase.

The relaxation intervention aimed to isolate the effects of the language intervention and help rule of alternative explanations for the experimental results. As such, the relaxation intervention was similar to the language intervention in that it involved taking part in a research study with multiple study visits involving various forms of testing before and after the intervention period, social interaction with new people in a group setting and, importantly, the expectation of receiving an intervention with potential benefits for cognition. The latter was achieved by informing participants that both the language course and the relaxation course constituted interventions with potential benefits for cognition in older adults. As such, participants were aware of the existence of the two treatment conditions but were blind to the research hypotheses.

The target sample size for the magnetic resonance (MR) component of the study was determined based on the statistical power required for detecting the differential change in gray matter from pretest to posttest in the language group relative to the relaxation group. As such, power calculations were performed for a mixed analysis of variance, testing for an interaction between the within-subject factor of time (pretest, posttest) and the between-subject factor of group (language, relaxation), using G*Power 3.1 (Faul et al., 2007). At an α-level of 0.05 and assuming high correlations for repeated measures for cortical thickness and hippocampal volume (*r* = 0.80), 82 participants were required to detect an interaction of a small magnitude (*d* = 0.2/*f* = 0.1) with a power of 0.80. Effect sizes lower than this were not considered to be of practical relevance. With a final sample size of 72 participants for the MR measures and pre-post correlations of 0.90, the achieved statistical power to detect a group by time interaction of *f* = 0.1 at *α* = 0.05 was high at 96%.

**Figure 1:**
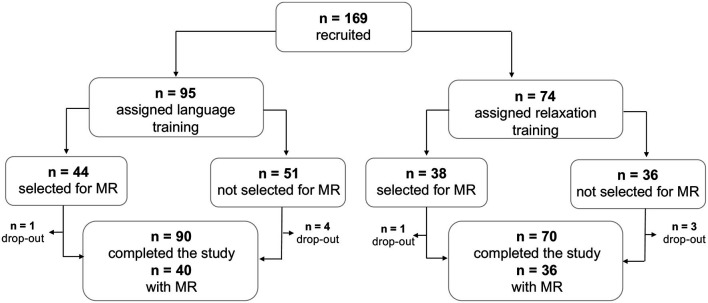
Study sample profile. Flowchart showing the conversion of the number of recruited participants to the number of participants who completed the study, with and without complete MR data.

A total of 169 participants fulfilled the study criteria and were recruited, of which 160 participants subsequently completed the study (Figure 1). Participants who expressed an interest, were right-handed and did not have any MR contraindications were selected to undergo the MR assessment. A total of 82 participants were assigned MR, of which 76 completed the assessment both at pretest and at posttest (Figure 1). Age, sex, and associative memory performance at pretest were comparable for the full sample and the MR subsample, supporting the representativeness of the subsample (Table 1). The study was approved by the ethical review board in Stockholm (Regionala Etikprövningsnämnden, Stockholm, case number 2015/2284-31/2) and conducted in accordance with the Declaration of Helsinki.

**Table 1:** Demographic information of the study sample.

	Language group		Relaxation group	
	All (*n* = 90)	MR (*n* = 40)	All (*n* = 70)	MR (*n* = 36)
Age (years)	69.24 (2.70)	69.35 (2.68)	69.49 (2.85)	69.89 (2.94)
Sex (f/m)	52/38	23/17	48/22	24/12
Associative memory	0.55 (0.26)	0.56 (0.27)	0.52 (0.26)	0.46 (0.26)

*Demographic information for the full study sample and the subsample that completed the MR assessment at pretest and posttest. Means (standard deviations) are presented for age, sex, and performance on the word-word associative memory test before the intervention, which were the variables used as stratifiers for randomization*.

### Study Design and Procedure

The study procedure included a pretest phase, an intervention phase, and a posttest phase. As such, the study employed a 2 (intervention: language vs. relaxation) × 2 (time: pretest vs. posttest) mixed factorial design with random assignment to intervention.

Both interventions lasted for 11 weeks and were administered in a group setting of maximally 12 participants at an adult education center in central Stockholm, which provided licensed teachers, course materials, and facilities. Participants in the language group received two 2.5 h long teacher-led classes per week (total of 55 h), which followed a coursebook with a pace of approximately one chapter per week. Every chapter included a main text (a dialogue), which served as the basis of the verbal communication exercises that were focused on during the classes. Basic grammatical information and a list of words and their Swedish translation accompanied the main text. The teacher-led classes had a strong focus on verbal communication, involving exercise dialogues, in groups and in pairs, of ordinary events connected with leisure or tourism (e.g., ordering coffee at a restaurant, asking for directions). In addition, as weekly homework, participants were asked to learn on average 44 (SD = 11) new Italian words (total of 437 words) in their own time, with learning success being assessed with a written test every week. The standard content of the beginners’ course and the accompanying coursebook was an application of the first (A1) level of the common European framework of reference for languages. Participants in the relaxation condition received one 1-h long teacher-led class per week, which focused on breathing techniques and relaxation exercises. To this end, participants laid on the floor as they listened to and followed the teacher’s instructions regarding breathing and relaxation.

Cognitive performance was assessed with an extensive test battery 2 weeks before (pre-test) and 1 week after the interventions (post-test). The cognitive tests and related results have been described in detail elsewhere (Berggren et al., 2020). Here, we focus only on the tests of direct relevance for the posed hypotheses. Achieved vocabulary proficiency was assessed with a written vocabulary test at the end of the language course. In contrast to the weekly vocabulary homework, participants were not aware that this final vocabulary test would be administered. The test consisted of 110 words randomly sampled from the chapters completed during the course. Participants were presented with the Swedish words in writing and were asked to write out the Italian translations. Participants were given a maximum of 2 points per correct word (correct spelling), 1 point if any minor error was present (e.g., correct word but spelling error) and 0 points for major error (e.g., incorrect word) or absent answer. The resulting maximum score was 220 points. There was no time limit for the final vocabulary test. Attendance and average performance on completed weekly homework tests were furthermore examined for descriptive purposes.

Associative memory was assessed with three item-associative memory tasks that varied in regards to the stimuli used for the associations to be remembered: word-word associations, face-name associations, or picture-picture associations. Each test consisted of three phases; encoding, item recognition, and associative recognition. In the encoding phase, participants were instructed to memorize 40 stimuli pairs, presented one pair at a time. Each presentation lasted 6,000 ms. During the subsequent item recognition phase, which was not of interest here, participants were presented with one stimulus at a time and asked to indicate whether they had seen that item before, as part of the item pairs during the encoding phase. During the associative recognition phase, participants were presented with stimuli pairs and asked to indicate whether they had seen that particular item pair during the encoding phase. The associative recognition task consisted of 20 stimuli pairs that had been seen before (target pairs) and 20 previously unseen stimuli pairs (foil pairs). In the foil pairs, each individual item had been presented during encoding, but not together in a pair. Across the three task versions, participants were therefore required to encode and later recognize a total of 120 stimuli pairs. Performance in the associative memory tasks was defined as the proportion of hits minus the proportion of false alarms in an effort to account for response bias.

### Magnetic Resonance Imaging

#### Acquisition and Pre-processing

One week before and 2 weeks after the intervention, MR imaging was performed with a GE Discovery MR-750 3.0-T scanner (General Electric, Milwaukee, WI, USA), located at the Karolinska University Hospital, in Solna, Sweden. An eight-channel head coil was used. Structural images were acquired with a standardized T1 spoiled gradient BRAVO sequence with 0.94 mm^3^ isotropic voxels, a field of view of 240 mm (240 × 240 matrix), repetition time/echo time = 6.4/2.808 ms, and flip angle 12°. Diffusion measures were acquired employing an echo planar imaging (EPI) sequence in 70 directions (*b* = 1,000 s/mm^2^) with 2.29 mm^3^ voxels, 62 axial slices, a field of view of 220 mm (220 × 220 matrix), repetition time/echo time = 7,800/90.9 ms, plus two volumes without diffusion weighting. The total acquisition time for the diffusion was 9.5 min.

**Figure 2:**
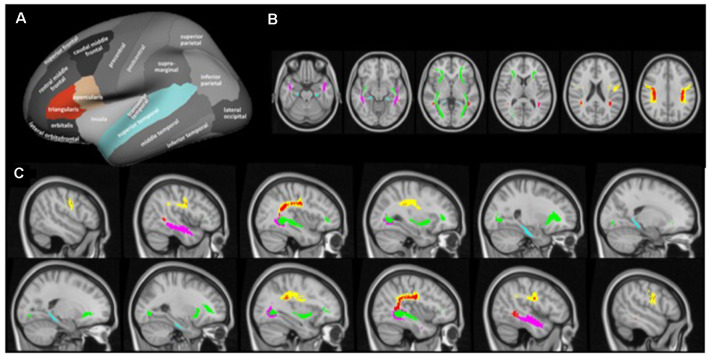
Cortical regions of interest **(A)** and white matter tracts of interest from axial **(B)** and sagittal direction **(C)**. Cortical regions of interest included the pars triangularus (red) and the pars opercularis (beige), both representative of the inferior frontal gyrus, and the superior temporal gyrus (blue), as indicated by the colored parcels in the Desikan-Killiany parcellation map (image modified with permission from Desikan et al., 2006). Note that the hippocampus was included as a region of interest but as a subcortical region is not depicted here. White matter tracts of interest included the superior longitudinal fasciculus (SLF; yellow), temporal component of the SLF (red), inferior longitudinal fasciculus (ILF; pink), inferior fronto-occipital fasciculus (green), and cingulum hippocampal (HC) part (blue), as defined in the JHU DTI-based white-matter atlas (Wakana et al., 2007), displayed on axial (MNI = 50, 60, 71, 82, 93, 103) and sagittal slices (MNI = ± 19, 25, 32, 38, 45, 51).

Cortical reconstruction and volumetric segmentation of the T1-weighted images were performed using the FreeSurfer imaging analysis suite^3^ (version 6.0). To extract reliable volume and thickness estimates, images were automatically processed with the longitudinal stream in FreeSurfer (Reuter et al., 2012). The longitudinal stream creates an unbiased within-subject image from the two time points (Reuter and Fischl, 2011), increasing reliability and statistical power compared to the regular FreeSurfer stream. Measures of cortical thickness were extracted for the hypothesized gray matter regions using the standard cortical parcellation in Freesurfer (Desikan et al., 2006). Specifically, average cortical thickness was extracted for each participant from the pars triangularis and the pars opercularis, both representative of the inferior frontal gyrus, and the superior temporal gyrus, in the right and left hemisphere (Figure 2A). Hippocampal (HC) volumes in the left and right hemispheres were extracted from the subcortical FreeSurfer segmentation and adjusted for total intracranial volume (ICV) as follows:

adjusted HC volume=raw HC volume−b X(ICV−average ICV),

where b is the slope of regression of the raw HC volume on ICV.

Following a visual quality check of the segmentations, data for four participants were excluded from analyses due to excessive motion at one of the time points (one in language group, one in relaxation group) or due to incomplete pre-processing likely caused by large anatomical deviations (one in language group, one in relaxation group). The remaining 72 participants (38 in the language group, 34 in the relaxation group) demonstrated good data quality at both time points and were included in the analyses.

Voxelwise statistical analysis of the fractional anisotropy (FA) and mean diffusivity (MD) data in the white matter tracts of interest was carried out using Tract-Based Spatial Statistics (TBSS, Smith et al., 2006), part of the FSL imaging analysis suite. FA and MD images were first created by fitting a tensor model to the raw data using FDT, and then brain-extracted using BET (Smith, 2002). The FA images of all subjects were then aligned using the nonlinear registration tool FNIRT. A mean FA image was then created and thinned to arrive at a mean FA skeleton which represents the center of all tracts common to the group. Each subject’s aligned FA data was then projected onto this skeleton and the resulting data fed into voxelwise cross-subject statistics. MD images were processed by applying the same nonlinear registration and skeletonization stages as for the FA images to the mean of the three eigenvalues. Out of the 76 scanned participants, no DTI was acquired from one participant and only at pretest for one participant (both in language group), and pre-processing did not complete for two participants (one in the language group, one in the relaxation group), leaving 72 participants with DTI data available at both time points. Visual inspection of the individual FA data projected onto the mean FA skeleton revealed good quality for all 72 participants included in analyses.

#### Statistical Analysis

The average cortical thickness in the pars triangularis, pars opercularis and the superior temporal gyrus, and hippocampal volumes adjusted for total ICV were exported to and analyzed in SPSS (version 25). Repeated-measures analyses of variance (ANOVA) were conducted for each region of interest separately, with group (language training, relaxation) as between-subject variable and time (pre, post) and hemisphere (left, right) as within-subject variables, restricting confirmatory analyses to the hypothesized group x time interaction. Effect sizes and their confidence intervals were derived using the metafor package in the R environment (Viechtbauer, 2010; R Core Team, 2017). As recommended (Becker, 1988), the difference in standardized mean change from pretest to posttest for the language group and the relaxation group was calculated for each region of interest using raw-score standardization.

$$g=g_{language}-g_{relaxation}$$

where

$$g_{language}=\left(c(n_{language}-1)\frac{{\bar{x}}_{post,language}-{\bar{x}}_{pre,language}}{SD_{pre,language}}\right)$$

$$g_{relaxation}=\left(c(n_{relaxation}-1)\frac{{\bar{x}}_{post,relaxation}-{\bar{x}}_{pre,relaxation}}{SD_{pre,relaxation}}\right)$$

where ${\bar{x}}_{post,language}$ and ${\bar{x}}_{pre,language}$ are the means at posttest and pretest for the language group, *SD_pre,language_* is the standard deviation of the pre-test scores, *c* (*n* − 1) is a bias-correction factor (equation 5 in Morris, 2000), *n_language_* is the sample size of the language group and ${\bar{x}}_{post,relaxation}$, ${\bar{x}}_{pre,relaxation}$, *SD_pre,relaxation_* and *n_relaxation_* are the analogous values for the relaxation group. The sign for *g_language_* and *g_relaxation_* was assigned so that a positive value represented a positive change in cortical thickness or volume. A positive value for *g* therefore indicated greater increases or smaller reductions in the language group, relative to the relaxation group. Sampling variance was estimated using measured pre-post correlations (Supplementary Material 2) with Equation 13 in Becker (1988). Confidence intervals for the effect sizes were obtained by fitting a fixed effects model using the metafor package (“rma” function).

To test the hypothesized association between gray matter change and vocabulary learning, change scores (posttest values − pretest values) were correlated with vocabulary test performance in the language group. Performance on the vocabulary test at posttest can here be considered to indicate a change in vocabulary because all individuals lacked proficiency in Italian at baseline. The statistical threshold for each analysis was corrected for the number of regions considered (α= 0.05/4 = 0.0125).

All statistical tests for FA and MD were performed using Randomise in FSL (version 6.0), employing 5,000 permutations (Winkler et al., 2014). The statistical threshold was set at *p* < 0.05 (FWE corrected), and the threshold-free cluster enhancement method was used to define the clusters. All hypotheses were tested using voxelwise statistical analysis within the tracts of interest, using a binary mask that combined the five relevant tracts in the JHU DTI-based white-matter atlas (Wakana et al., 2007), intersected with the mean FA skeleton. The five tracts of interest were SLF, temporal component of the superior longitudinal fasciculus (SLFt), ILF, inferior fronto-occipital fasciculus (IFO), and cingulum hippocampal part (cHC; Figures 2B,C). To test the hypothesized group × time interaction, difference images were calculated for each participant by subtracting the posttest 4D image from the pretest 4D image and a two-sample sample unpaired *t-test* was used to test the effect of group on these images. The same difference images were used to test the hypothesized correlation between change in white matter microstructure and vocabulary test performance in the language group. Finally, pretest images were correlated with vocabulary test performance in the language group.

Structural equation modeling (SEM) was used to test how associative memory and hippocampal volume, both modeled in latent space, predict vocabulary learning success in the language-learning group. Analyses were performed using the lavaan package in the R programming environment, employing the structural equation modeling function (“sem”) to fit the model and maximum likelihood to estimate the parameters (Rosseel, 2012; R Core Team, 2017). Associative memory was modeled as the shared variance among three associative recognition tests and the hippocampal volume was modeled as the shared variance of adjusted volumes in the left and the right hemisphere, all at pretest. In a confirmatory model, which followed the hypothesis directly, performance on the vocabulary test at the end of language training was regressed on the latent variables of associative memory and hippocampal volume, and the predictors were allowed to correlate.

Data from participants in the language training group was used to estimate the parameters in the model. As such, the total number of cases was equivalent to the number of participants in the language group (*n* = 90), with data available for all 90 participants for all three associative memory tests and the final vocabulary test. MR images of sufficient quality were available for 38 of the participants. All observed variables were standardized before being entered into the model. Full-information maximum likelihood estimation that was used to treat missing data. Model fit was evaluated using comparative fit index (CFI) and RMSEA and a statistical threshold of 0.05 was used to determine the significance of the estimated associations in the models. Although the age range of the tested sample was relatively narrow, both cognitive function and structural brain integrity can be expected to change between the age of 65 and 75 years. A sensitivity analysis was therefore subsequently performed using age as an additional predictor.

## Results

### Descriptive Results

**Figure 3:**
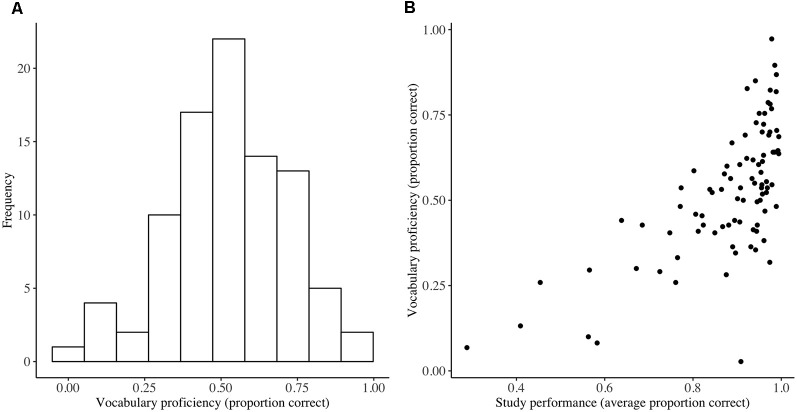
Vocabulary test performance **(A)** and its relationship with study performance **(B)**. Distribution of performance on the final vocabulary test at the end of language training (vocabulary proficiency) and a scatterplot demonstrating the association between vocabulary proficiency and average performance on the weekly homework (*n* = 90).

Attendance at the language course was high, with an average number of 20.16 sessions attended out of the possible 22 (SD = 1.57, range = 17–22). The average proportion correct on the weekly homework was 0.88 (SD = 0.14) for initiated tests, with a negatively skewed distribution. Performance on the final vocabulary test was lower with an average proportion correct of 0.54 (SD = 0.19; Figure 3) Performance on the final vocabulary test was positively correlated with average performance on the weekly homework (Kendall’s rank correlation; τ*b* = 0.49, *p* < 0.001; Figure 3), which was expected given that the words in the final test were studied as part of the homework.

Of relevance to the structural equation model, in the language group, associative memory performance for face-name pairs (*M* = 0.33, SD = 0.19), word-word pairs (*M* = 0.55, SD = 0.26), and picture-picture pairs (*M *= 0.47, SD = 0.25) were all moderately correlated at pretest (all *r* > 0.36, all *p* < 0.001). Similarly, adjusted hippocampal volumes in the left and right hemispheres were highly correlated in the language group at pretest (*r* = 0.71, *p* < 0.001). The language group and relaxation groups were well matched in regards to basic demographics, gray matter, and cognitive measures at baseline (Supplementary Material 1).

### Hypothesis Testing

#### Language Training in Older Adults, Relative to Relaxation Training, Will Result in Gray Matter Changes in the Following Language-Relevant Areas: the Inferior Frontal Gyrus, Superior Temporal Gyrus, and the Hippocampus

**Figure 4:**
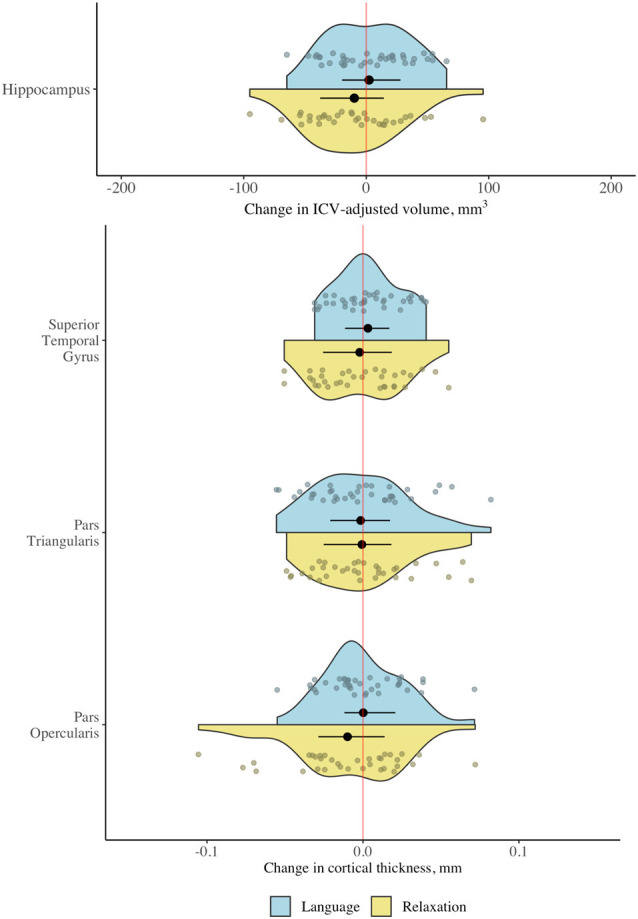
Change in gray matter regions of interest following language and relaxation training. Distribution of change scores (post − pre) for cortical thickness and adjusted hippocampal volume in the language group (*n* = 38) and the relaxation group (*n* = 34). Black dots and line segments denote the mean and 1st and 3rd quartiles. Smaller dots denote individual change scores. Note that the scale is different for cortical thickness (mm) and volume (ICV-adjusted mm^3^).

Repeated-measures analyses of variance revealed no significant interaction between group (language vs. relaxation) and time (pretest vs. posttest) for cortical thickness in any of the gray matter regions of interest at the corrected (α = 0.0125) or the uncorrected statistical threshold (α = 0.05), providing no evidence for differential gray matter change in the two groups for any of the regions of interest (Table 2; for full ANOVA output see Supplementary Material 3; for descriptive statistics see Supplementary Material 2). Effect size estimates, expressed as the difference between the standardized mean change in the two groups, were small (Table 2). There were also no significant main effects or any other interaction effects at the corrected statistical threshold (Supplementary Material 3). The distribution of the raw change scores is presented in Figure 4, which demonstrates the great overlap between the language and relaxation groups, both of which center at zero, for all regions of interest. Pre-post correlations were very high for all regions in both groups, evidencing good reliability of the measures (all *r* values >0.92; Supplementary Material 2). An exploratory whole-brain voxel-based morphometry (VBM) analysis revealed no significant voxels for the hypothesized interaction effect (Supplementary Material 4).

**Table 2:** Interaction effect of group and time on gray matter in regions of interest.

	Group x Time			Effect size	
Region of interest	F	df	*p*	g	95% CI
Inferior Frontal Gyrus, pars opercularis	1.923	1	0.170	0.107	−0.052, 0.266
Inferior Frontal Gyrus, pars triangularis	0.012	1	0.912	−0.010	−0.175, 0.154
Superior temporal gyrus	0.863	1	0.356	0.054	−0.065, 0.172
Hippocampus	0.771	1	0.383	0.041	−0.056, 0.138

*Interaction between group (language, relaxation) and time (pre, post) derived from repeated-measures analyses of variance for cortical thickness (pars opercularis, pars triangularis, superior temporal gyrus) and volume adjusted for ICV (hippocampus). Effect sizes represent the difference in standardized mean change (pre-post) in the language group and the relaxation group (language-relaxation)*.

#### Gray Matter Changes in the Inferior Frontal Gyrus, Superior Temporal Gyrus, and the Hippocampus Will be Associated With Vocabulary Proficiency at the End of Language Training

Correlation analyses revealed no significant correlations between change in any of the gray matter regions of interest and vocabulary proficiency at the end of language training (Table 3). Vocabulary proficiency and all gray matter variables that were entered into correlational analyses were approximately normal. An exploratory whole-brain voxel-based morphometry (VBM) analysis revealed no significant voxels for the correlation between gray matter volume change and vocabulary proficiency at the end of language training (Supplementary Material 4).

**Table 3:** Relationship between achieved vocabulary proficiency and change in gray matter regions of interest.

Variable	Skew (SE)	Kurtosis (SE)	Correlation with vocabulary proficiency (r)	*p*
Inferior Frontal Gyrus, pars opercularis (change, mm)	0.440 (0.383)	0.576 (0.750)	−0.181	0.277
Inferior Frontal Gyrus, pars triangularis (change, mm)	0.498 (0.383)	0.202 (0.750)	−0.159	0.341
Superior temporal gyrus (change, mm)	0.146 (0.383)	−0.905 (0.750)	−0.297	0.070
Hippocampus (change, mm^3^, adjusted)	0.120 (0.383)	−0.712 (0.750)	0.060	0.721
Vocabulary proficiency (prop correct)	−0.264 (0.254)	0.209 (0.503)	-	-

*Correlations between gray matter change from pretest to posttest in regions of interest, across hemispheres, and vocabulary proficiency at the end of language training (*n* = 38). Corrected α = 0.0125*.

No predictions were made concerning lateralization but exploratory correlational analyses revealed no significant correlations in any hemisphere at the corrected statistical threshold (Supplementary Material 5). Furthermore, with the exception of the hippocampus, no predictions were made regarding the gray matter at pretest predicting vocabulary proficiency in the language group, but exploratory analyses revealed no significant correlations at the corrected statistical threshold (Supplementary Material 6).

#### Language Training in Older Adults, Relative to Relaxation Training, Will Result in a Differential Change in White Matter Microstructure in Language-Relevant White Matter Tracts

No voxels demonstrated significance under the specified statistical threshold for the effect of group (language vs. relaxation) on pre-post change in FA or MD, anywhere within the tracts of interest. An exploratory whole-brain analysis revealed no voxels with a significant effect of group on pre-post change in FA or MD (Supplementary Material 4).

#### Changes in White Matter Microstructure in Language-Relevant White Matter Tracts Will Be Associated With Vocabulary Proficiency at the End of Language Training

No voxels demonstrated a significant association between vocabulary learning success and pre-post change in FA or MD in the language group, anywhere within the tracts of interest.

An exploratory whole-brain analysis revealed two voxel clusters showing a significant positive correlation between change in MD between pre-test and post-test and vocabulary proficiency at the end of language training, both with peaks in the genu of the corpus callosum (Supplementary Material 4). No significant voxels were found for the correlation between change in FA between pretest and posttest and vocabulary proficiency (Supplementary Material 4).

#### The White Matter Microstructure in Language-Relevant White Matter Tracts at Baseline Will Be Associated With Vocabulary Proficiency at the End of Training

No voxels demonstrated a significant association between vocabulary learning success and FA or MD at pretest in the language group. An exploratory whole-brain analysis revealed no voxels with a significant correlation between FA at pre-test and vocabulary proficiency at the end of language training (Supplementary Material 4).

#### Associative Memory Ability and Hippocampal Volume at Pretest Predict Shared as Well as Unique Aspects of Vocabulary Proficiency at the End of Language Training

**Figure 5:**
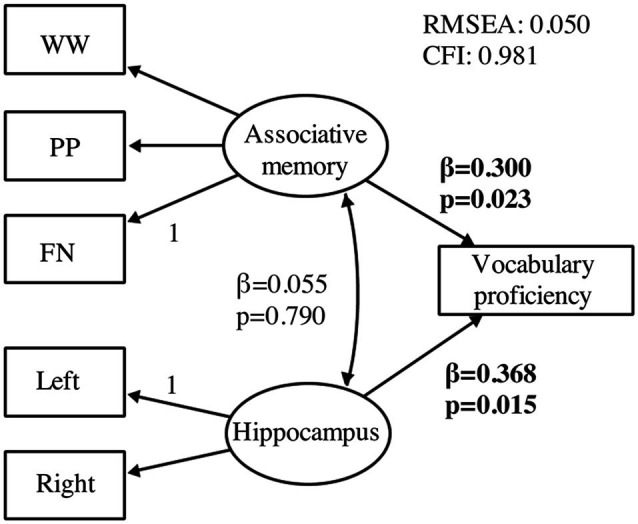
Confirmatory structural equation model testing predictors of vocabulary proficiency. The model tests associative memory (*n* = 90) and hippocampal volume (*n* = 38) at pretest as predictors of vocabulary learning at the end of language training (*n* = 90). All estimates are standardized and effects with *p* < 0.05 are presented in bold font. Regression weights are represented by single-headed arrows and covariances by double-headed arrows. Regression weights marked with a 1 were restricted to 1. All other regression weights, covariances, and intercepts were estimated. WW, word-word; PP, picture-picture; FN, face-name.

The confirmatory structural equation model demonstrated a good fit and revealed that both associative memory and hippocampal volume significantly predicted vocabulary proficiency following language training (Figure 5). However, the model provided no evidence that hippocampal volume was associated with associative memory ability at pretest. The sensitivity analysis, in which age was included as an additional regressor, did not change the outcome of the analysis.

## Discussion

The present study is the first to investigate the effect of language training on brain structure in older adults. Despite good course attendance and demonstrated foreign vocabulary acquisition, there was no evidence for structural change in language- and memory-relevant gray and white matter regions following an entry-level language course, relative to a relaxation course of the same duration. However, hippocampal volume and associative memory ability were identified as robust predictors of achieved vocabulary proficiency at the end of language training.

Hippocampal volume has previously been found to predict performance on several learning and recall paradigms, but this has not been consistently found when healthy older individuals are considered separately from individuals with Alzheimer’s disease (AD), mild cognitive impairment, or probable AD (Petersen et al., 2000; Walhovd et al., 2010). One reason for this may be that most previous studies have used short retention intervals, most often less than 1 h, between learning trials and the memory test (Fjell et al., 2005). Walhovd and colleagues (Walhovd et al., 2004) studied healthy individuals and found an age-independent relationship between hippocampal volume and free recall for a retention interval of several weeks but not of 30 min, supporting the idea that hippocampal size may be particularly important for the ability to retain information over long delays. The present study gives further support to this conclusion, identifying hippocampal volume as a predictor of memory for information that had to be retained for 1 to 10 weeks from the time that it was encoded. Associative memory was also identified as a predictor of vocabulary proficiency, which was expected given the likely dependence on this cognitive ability for encoding and retrieving the Italian-Swedish word combinations in the weekly homework. Importantly, like hippocampal volume, associative memory was modeled in latent space, as the shared variance among three associative recognition tests, which enabled us to reduce task-specific effects in favor of effects that are general to associative memory ability. Thus, consistent with our hypotheses, having large hippocampi and good associative memory appear to be beneficial for foreign word learning in healthy older adults.

In contrast to our hypothesis, however, hippocampal volume and associative memory ability were not significantly associated at pretest, which seemingly contradicts the role of the hippocampus in associative memory (Suzuki, 2008). However, evidence for the importance of hippocampal volume per se for associative memory has not been consistent. A meta-analysis reported only a weak positive association between hippocampal volume and episodic memory in older adults and findings relating specifically to associative memory in older adults have been mixed (Becker et al., 2015; Zheng et al., 2017). Also, in relation to the previous discussion, it is possible that the short retention interval in the associative recognition tests may have limited the role of hippocampal volume in the present study. Furthermore, the hippocampus is not a unitary structure and certain subfields within the hippocampus are thought to be particularly important for associative and episodic memory (Carr et al., 2017). Here we did not have the required resolution to investigate hippocampal subfields, making it possible for a potential weak association to have been averaged out. Another consideration is that relative to manual tracings, the FreeSurfer segmentation algorithm is known to overestimate hippocampal volumes, more so in older populations, even though this bias is thought to be reduced in the later FreeSurfer version used here (version 6.0.0; Wenger et al., 2014; Schmidt et al., 2018). Whilst such a bias calls for caution in interpreting the absolute hippocampal volumes reported here, the present study concerns conclusions across a relatively narrow age span (65–75 years), which are less vulnerable to the skew that the bias could introduce for age-related interpretations. Whilst hippocampal volume was found to predict vocabulary learning success, white matter microstructure before language training, as indexed by FA and MD, did not predict learning anywhere in the SLF, ILF, inferior fronto-occipital fasciculus (IFO) and the hippocampal section of the cingulum, which was inconsistent with our hypothesis and some previous research (Qi, 2015). The apparent discrepancy of the result in the hippocampus and in white matter can be considered in the context of test requirements. The final vocabulary test required free recall and subsequent written production of previously encoded foreign-native word pairs, which is likely to have involved recall from the mental lexicon but likely missed more procedural aspects of language processing, such as grammatical knowledge and spoken language processing (Ullman, 2004). As such, the hippocampus may have been more important for vocabulary learning as it was tested here, relative to the white matter tracts that predominantly connect classical language regions.

Contrary to the hypotheses, the study found no evidence that the language intervention triggered a structural change in the hippocampus or in any of the other specified language- and memory-relevant gray and white matter regions. Specifically, no significant differential change could be detected in cortical thickness in the inferior frontal gyrus or the superior temporal gyrus, or in hippocampal volume, following the language course, relative to a relaxation course of the same duration. Effect size estimates indicated that any group differences in change in cortical thickness in the pars opercularis (*g* = 0.107, 95% CI = −0.052, 0.266), the pars triangularis (*g* = −0.010, 95% CI = −0.175, 0.154), the superior temporal gyrus (*g* = 0.025, 95% CI = −0.065, 0.172) and in hippocampal volume (*g* = 0.041, 95% CI = −0.056, 0.138) are unlikely or likely to be small. In regards to white matter microstructure, no significant differential change was detected in FA or MD using a voxel-wise approach, anywhere in the SLF, ILF, inferior fronto-occipital fasciculus (IFO) and the hippocampal section of the cingulum. The degree of change in these gray matter and white matter regions was furthermore not significantly associated with achieved vocabulary proficiency at the end of language training. An exploratory whole-brain analysis revealed a positive association between change in MD from pretest to posttest and vocabulary proficiency at the end of language training in the genu of the corpus callosum. This incidental finding points to the possibility that changes in white matter microstructure outside of the traditional language- and memory-relevant tracts considered here could be of greater importance for vocabulary learning. Whilst this possibility should be considered in future research, we restrict our conclusions to the regions specified *a priori*, for which the language intervention did not result in any detectable morphological brain change, independent of the level of achieved vocabulary proficiency. Several possible explanations should be considered when interpreting these results, as discussed below.

It is possible that the language intervention was insufficient for triggering a reliable structural change in the present study. In regards to the extent of the language input, it has been postulated that a long-lasting mismatch between supply and demand is necessary for triggering experience-dependent structural plasticity (Lovden et al., 2010). Whilst such a theoretical formulation is inherently difficult to translate into absolute terms, previous investigations have been able to detect structural brain changes following language interventions of similar duration and intensity in younger adults (e.g., 10-week long intervention in Bellander et al., 2016; 46 h of study over 16 weeks in Hosoda et al., 2013). In particular, the language intervention employed by Bellander et al. (2016) shared several aspects of the one used here: both interventions involved the same entry-level Italian course, which was delivered by the same teachers at the same adult education center (Bellander et al., 2016). The interventions differed in that the present intervention included more teacher-led classes over a longer period (5.0 h per week for 11 weeks vs. 2.5 h per week for 10 weeks), different performance on the final vocabulary exam (54% correct in the present study vs. 27% correct) and different format and extent of the vocabulary learning during the intervention period (maximum 437 words *via* traditional glossary learning in the present study vs. maximum 2,300 words *via* a mobile phone app). In particular, the greater number of words to be learned in the study by Bellander et al. (2016) is likely to have posed a greater challenge and may therefore account for structural change being detected in the hippocampus in that study and not in the present one.

Another aspect that could account for the differential findings in the present study and the study by Bellander et al. (2016) concerns the age of the study samples. Normal aging has been linked to several neural changes that affect plasticity negatively, particularly in hippocampal and frontal regions (Burke and Barnes, 2006; Lovden et al., 2010). As such, the lack of structural change in the hippocampus in the present study, and in other regions of interest, may be due to insufficient plastic resources in older individuals. Alternatively, the intervention dosage may have to be modified to achieve the same structural change in old as in young individuals. It is worth noting, however, that experience-dependent structural changes have been demonstrated in response to similar dosage for older as for younger adults in non-language domains, indicating that it is not always necessary to modify dosage for the older population (Boyke et al., 2008; Lövdén et al., 2010; Wenger et al., 2012). In fact, it may be argued that an intervention with the same objective demand may be subjectively more demanding, and thus serve as a higher impetus for plastic changes, for older than for younger adults (Lovden et al., 2010). Another aspect that may have influenced the relative dosage of the language intervention is the fact that participants were not strictly monolinguals but had at least a working level in English, which may have limited the effort required for the introductory Italian course for the native Swedish speakers in the present study, potentially reducing the need for structural neuroplastic changes.

In regards to the lack of reliable change in gray matter and white matter, it is also possible that the qualitative content of the language intervention was not optimally targeted to trigger a structural change in regions with particular relevance for language processing. The intervention involved teacher-led exercises that aimed to promote verbal communication, pronunciation, grammar, and vocabulary, but other higher-level language skills, such as abstraction and generation of a new language, were necessarily beyond the scope of the entry-level course. It may be that aspects such as continuous practice and immersion in bilingual environments are necessary to achieve a level of language skill that has neuroprotective effects (Del Maschio et al., 2018). In regards to the active control condition, the relaxation intervention included and thereby controlled for aspects of multiple study visits, social interaction in a group setting, and an expectation of receiving an intervention with benefits for cognition, but was otherwise relatively loosely matched to the language intervention. Whilst it is important to acknowledge that the relaxation intervention, therefore, is unlikely to have controlled all potential confounds, this concern has less relevance for the null findings reported here but should be considered for improved control of false positives in future research. It is also worth considering that the MRI assessment was not performed immediately after the intervention period, which raises the possibility that any subtle structural changes following language training may have been reversed at the time of posttest scanning.

Another aspect is the potential effects of interindividual variation in learning during the intervention. Whilst interindividual differences in achieved vocabulary proficiency were found not to be related to change in gray or white matter, participants may have differed in other aspects. Since active participation in class exercises was encouraged but not enforced, it is possible that the quality of the intervention during classes differed amongst participants, which may have diluted potential effects. The assessment of language learning was unfortunately limited to vocabulary proficiency in the present study, which leaves the effect of interindividual differences in other aspects of language learning on brain structure an open question. Other than the nature of the intervention and the age of the sample, we cannot exclude the possibility that structural change may be occurring in regions and networks not directly investigated here, such as the cognitive control network, which has been implicated in studies of bilingual individuals (Luk et al., 2011; Abutalebi et al., 2012). The exploratory whole-brain analyses provided no indication that differential structural changes were taking place in the language group in areas outside the regions and tracts of interest, but caution is warranted in interpreting these null findings given their *post hoc* and under-powered nature. Future research will clearly be needed to determine whether the apparent lack of structural change following language training in the present study was due to insufficient demand or qualitative content of the language intervention, insufficient resources for plastic change in the older sample, an inappropriate search area, or all of the above.

From the present results, it can be concluded that a short-term entry-level language course did not result in any detectable structural changes in regions relevant for language and memory in older individuals, independent of the level of achieved vocabulary proficiency. Hippocampal volume and associative memory ability were both identified as robust predictors of vocabulary learning in older age. Future research should attempt to delineate to what extent the nature of the language input or the age of the sample may have hindered structural change from occurring.

## Data Availability Statement

The raw data supporting the conclusions of this article will be made available by the authors, without undue reservation.

## Ethics Statement

The studies involving human participants were reviewed and approved by Regionala Etikprövningsnämnden, Stockholm. The patients/participants provided their written informed consent to participate in this study.

## Author Contributions

ML developed the study concept. ML and RB designed the study. RB headed the data collection. JN, BG, and AL analyzed the data. JN drafted the manuscript. All authors contributed to the article and approved the submitted version.

## Conflict of Interest

The authors declare that the research was conducted in the absence of any commercial or financial relationships that could be construed as a potential conflict of interest.

## Footnotes

^1^https://osf.io/nv89a/register/564d31db8c5e4a7c9694b2c0

^2^https://osf.io/f3yu4/register/564d31db8c5e4a7c9694b2c0

^3^https://surfer.nmr.mgh.harvard.edu
