# Supplementary material for: Second Language Learning in Older Adults: Effects on Brain Structure and Predictors of Learning Success
Source: Front Aging Neurosci. 2021 Jun 3;13:666851. doi: 10.3389/fnagi.2021.666851 (PMC8209301; doi:10.3389/fnagi.2021.666851)
Supplement: Supplementary file 1 [file Table_1.DOCX]

Supplementary Material

## Supplementary material 1. Group comparison of age, sex and associative memory for word pairs. Descriptive statistics and comparisons for group differences at baseline for the measures that were used as stratifiers in randomization.

##

**Supplementary material 2.** Descriptive statistics for variables included in the repeated-measures ANOVA (S3). Means, standard deviations and pre-post correlations for grey matter regions of interest.

## Supplementary material 3. Full output from repeated-measures ANOVA. Main effects and interaction effects for the effect of time (pre, post), group (language, relaxation) and hemisphere (left, right) on grey matter in regions of interest.

**Supplementary material 4.** Exploratory whole-brain analyses.

For grey matter, analyses were conducted using modulated voxel-based morphometry (VBM) in SPM, using the cross-sectional pipeline in the CAT12 toolbox extension. In brief, using a flexible factorial design (Gläscher & Gitelman, 2008), the VBM analysis revealed no significant voxels for the group (language vs. relaxation) by time (pretest vs. posttest) interaction, neither in the expected direction (i.e. greater pre-post change in grey matter in the language group; p>0.05, FWE-corrected) nor in the unexpected direction (i.e. less pre-post change in grey matter in the language group; p>0.05, FWE-corrected). There were also no voxels that demonstrated significant correlations between grey matter changes (difference between pre and post images) and vocabulary proficiency at the end of language training and, in either direction (p>0.05, FWE-corrected).

For white matter, exploratory analyses were performed for FA and MD as previously described but with 500 permutations and with no mask other than the mean skeleton (see section 2.3.2. in manuscript). No voxels demonstrated significance for the effect of group (language vs. relaxation) on pre-post change in FA and MD, when considering the full skeleton (p>0.05, FWE-corrected). Similarly, no voxels demonstrated a significant association between FA or MD at pretest and vocabulary proficiencty at the end of language training (p>0.05, FWE-corrected). There were also no voxels that showed a significant association between vocabulary learning success and change in FA from pretest to posttest, in either direction (p>0.05, FWE-corrected). However, for MD, two voxel clusters demonstrated a positive association between change from pretest to posttest and vocabulary learning success (p<0.05, FWE-corrected), but no significant voxels for the negative association (p>0.05, FWE-corrected). The largest of the significant voxel clusters included 1700 voxels and had its peak in the genu of the corpus callosum (see supporting figure and table). Whilst this incidental finding must be interpreted with caution, it indicates that greater changes in mean diffusivity in the genu of the corpus callosum from before to after language training are associated with greater vocabulary proficiency at the end of the same training.


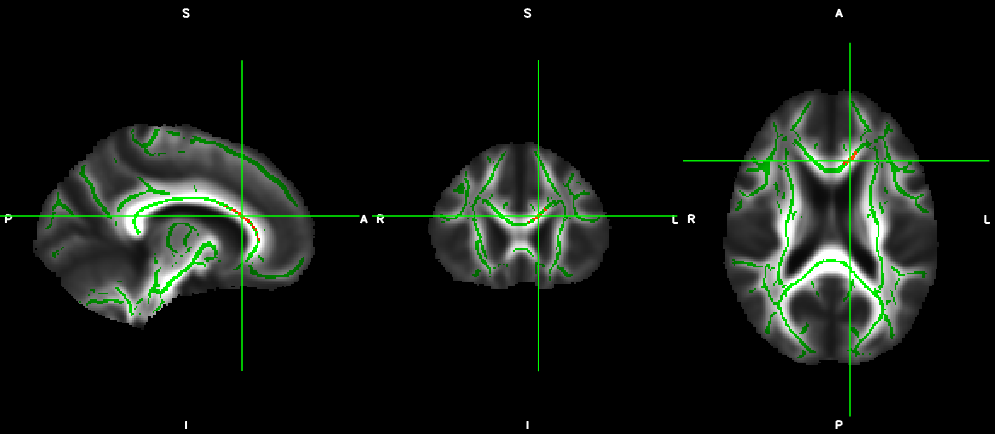


*Supporting figure for supplementary material 4.* Visualization of the largest voxel cluster (red) from the TBSS analysis that showed a significant association between change in MD from pretest to posttest and vocabulary learning success (p<0.05, FWE-corrected), overlaid on the mean FA skeleton (green), both displayed on the mean FA image (grey scale). The crosshairs are located at the peak voxel in the cluster (-10, 22, 18), in the genu of corpus callosum.

*Supporting table for supplementary material 4.* Tabulation of the significant voxel clusters from the TBSS analysis for the association between change in MD from pretest to posttest and vocabulary learning success, including cluster size, p-value, peak coordinates and center of gravity coordinates.

| cluster | voxels | p-value (FWE_corr_) | peak (X, Y, Z) | center of gravity (X, Y, Z) |
| --- | --- | --- | --- | --- |
| 1 | 1700 | 0.018 | -10, 22, 18 | -16, 32, 15 |
| 2 | 39 | 0.044 | -35, 37, 2 | -33, 36, 3 |

**Supplementary material 5.** Exploratory correlations between change in grey matter and vocabulary proficiency. Pearson’s correlations presented across and by hemisphere, in the language group.

**Supplementary Table 6.** Exploratory correlation analyses between pretest grey matter and vocabulary proficiency. Pearson’s correlations presented across and by hemisphere, in the language group.
